# Microbial Hydroxylation of 16α, 17α-Epoxyprogesterone by *Penicillium Decumbens*

**Published:** 2017

**Authors:** Shuhong Mao, Xuerong Wang, Zhijiang Ge, An Su, Lixia Zhang, Yanqing Li, Xiaoguang Liu, Fuping Lu

**Affiliations:** a *Key Laboratory of Industrial Fermentation Microbiology* *,* *Ministry of Education* *,* *Tianjin University of Science & Technology* *, * *Tianjin* *, * *China. *; b *Department of Chemistry and Chemical Biology, Rensselaer Polytechnic Institute, Troy, NY, USA. *; c *Tianjin Key Laboratory of Industrial Microbiology, College of Biotechnology, Tianjin University of Science & Technology, Tianjin, China.*

**Keywords:** Steroid, Biotransformation, *Penicillium decumbens*, 16α, 17α-Epoxyprogesterone, Hydroxylation

## Abstract

Microbial transformation has been successfully applied in the production of steroid intermediates with therapeutic use and commercial value in pharmaceutical industry due to its high regio- and stereo-selectivity. As such, it is still important to screen microbial strains with novel activity or more efficient abilities in the development of the commercial steroid industry. Biotransformation of steroid: 16α, 17α-epoxyprogesterone (1). using *Penicilliumdecumbens* as biocatalyst was investigated and selective hydroxylation of 1 was observed. The products were separated by silica gel column chromatography, and the structure determination was performed by MS, NMR, and X-ray crystallography. Biotransformation of 1 afforded 7β-hydroxy-16α, 17α-epoxyprogesterone (2). and 7β,11α-dihydroxy-16α,17α- epoxyprogesterone (3). The two novel metabolic products 2 and 3 were reported for the first time. Moreover, the identified C7β- and C11-αhydroxylation is a novel reaction of microbial transformation of steroids by *P.decumbens*.

## Introduction

Steroid, an important class of bioactive compounds, has been widely used as anti-inflammatory, anti-microbial, anti-diabetic, anti-allergenic, and anti-cancer drugs in clinical therapy (1-3). It is well known that the structural modification of steroid, such as the introduction of hydroxyl unit to different positions would change the activity and stability of steroid in different ways (4-6). Hence, there has been increasing interest in the synthesis and structural modification of steroid (4-6). Chemical synthesis has shown many disadvantages as a traditional method for the preparation of drug molecules such as the toxicity of chemical catalysts, complicated reaction steps, long production time, and low yield (7). However, biotransformation can overcome most of the shortcomings of chemical synthesis mentioned above, especially for the production of compounds with complex structures like steroids (8-10). In addition, the biotransformation is more efficient than chemical synthesis in reactions that require high steroselectivity or regioselectivity (11). 16α, 17α-Epoxyprogesterone (1) often serves as an important intermediate for many hormone based drugs such as hydrocortisone, cortisone, and megestrol (12). 

Although the biotransformation of (1) has already been successfully applied in industry for the production of 11α-hydroxylation16α, 17α-Epoxyprogesterone (3).the screening of microbial strains with novel catalytic activities still plays an important role in developing more efficient production processes as well as producing novel steroid compounds. For example, 7α-, 9α-, 11α-, 14α-, 11β- and 20β hydroxylation of (1) has been reported to be conducted by various filamentous fungi (3, 13).

In our study, *Penicillium decumbens* was observed to efficiently transform 16α, 17-epoxyprogesterone during the screening of fungal strains and two new hydroxylated steroid derivatives were obtained. Since *P. decumbens*is already known to have activity in the reduction of double bonds and hydroxylation of steroids (14, 15). The result of our study also indicates that *P. decumbens* can be applied to the production of novel hydroxylated steroids.

## Experimental

All chemical reagents were of analytical grade from commercial suppliers. TLC was conducted on a silica gel plate (Merck GFZZ34, 0.25 mm). With acetate ester/petroleum ester (1: 1, V/V). 

As asolvent. Spots were detected through UV-light (254 nm). ^1^H-NMR spectra were recorded on a JEOL JNM GSX 400M spectrometer in DMSO. ^13^C-NMR spectrawere measured at 100 MHz in DMSO. Mass spectra were performed on Waters 3100/2767with electron impact ionization (EI) at 70 eV. X-ray crystallography were carried out with Mo Karadiation (k = 0.07 nm) using a bruker APEX CCD diffractometer at 293 K.

**Figure 1:**
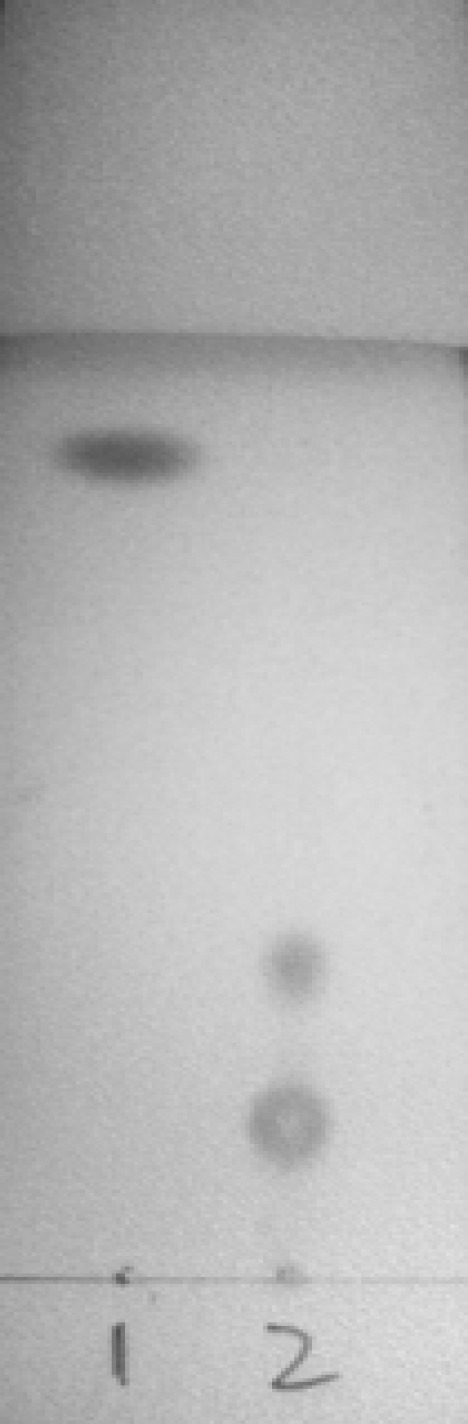
TLC analysis of transformation products by *P. decumbens*

**Figure 2:**
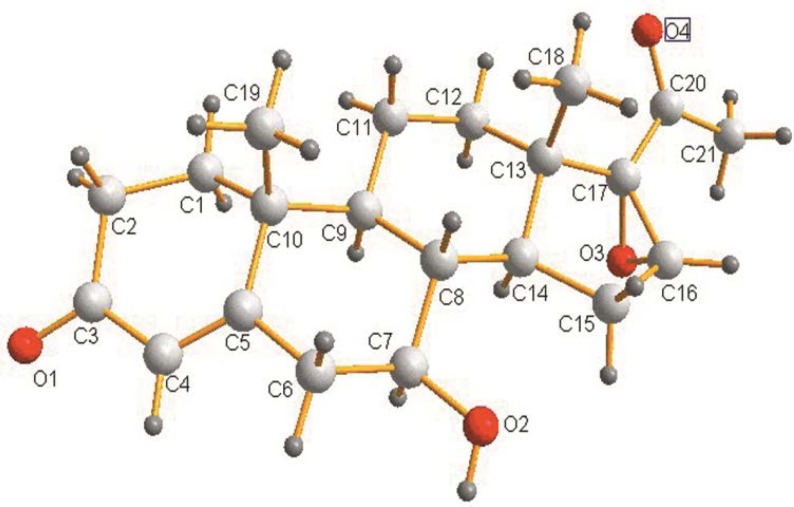
Crystal structure of 7β-hydroxy-16α, 17α-epoxyprogesterone (2)

**Figure 3:**
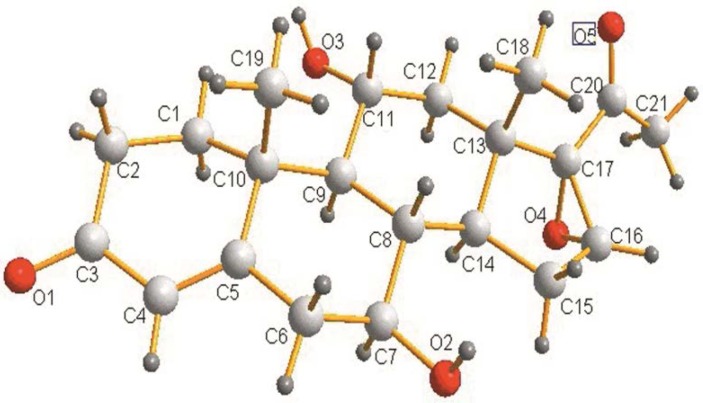
Crystal structure of 7β, 11α-dihydroxy-16α, 17α-epoxyprogesterone (3)

**Scheme 1:**
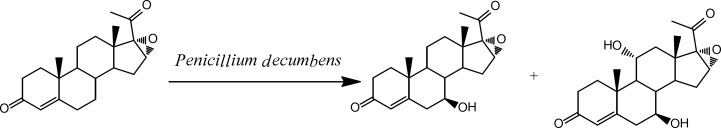
Biotransformation of 6α, 17α-epoxyprogesteroneby *P.decumbens*

**Table 1:** Characteristic chemical shifts in 1H NMR spectra of steroids（1）（2）（3） No. d (ppm).

**Substrate**	**δ (ppm)**			
	**H-C(7)**	**H-O(7)**	**H-C(11)**	**H-O(11)**
1	1.29	/	1.40	/
2	3.15-3.13	4.70	1.40	/
3	3.17-3.14	4.71	3.87-3.84	4.40

**Table 2:** Characteristic chemical shifts in 13C NMR spectra of steroids （1）（2）（3） No. d (ppm)

**No**.	**δ (ppm)**										
	C(1)	C(2)	C(3)	C(4)	C(5)	C(6)	C(7)	C(8)	C(9)	C(10)	C(11)
1	31.6	38.7	198.5	123.7	171.1	34.0	32.3	33.0	53.8	45.0	20.4
2	35.3	39.8	198.5	124.1	168.7	50.4	70.1	39.4	38.1	44.4	26.2
3	35.8	41.0	198.0	123.5	167.8	42.5	68.8	38.5	54.9	38.9	66.1

**Table 3:** Characteristic chemical shifts in 13C NMR spectra of steroids （1）（2）（3） No. d (ppm)

**No**.	**δ (ppm)**									
	C(12)	C(13)	C(14)	C(15)	C(16)	C(17)	C(18)	C(19)	C(20)	C(21)
1	27.2	35.4	31.7	26.2	60.6	70.6	15.4	19.0	205.3	17.2
2	34.1	39.6	31.4	30.3	61.1	73.5	15.3	20.4	205.5	17.2
3	39.1	38.7	33.3	29.2	60.3	72.1	15.2	25.1	204.3	17.5

The CCDC numbers of the crystal of 2 and 3were obtained after their crystallographic data wasdeposited with the Cambridge Crystallographic Data Centre. Further details ofthe crystallographic parameters canbe obtained for free on application to CCDC assupplementary publication, 12 UnionRoad, Cambridge CB2 1EZ, UK

*Microorganism cultivation and substrate biotransformation*

*P.decumbens *TCCC 41604, stored in our laboratory,was cultured on potato-dextrose-agar medium ina test tube at 28 °C for 4-5 d. 8 mL of sterilized water was added subsequently and 1ml of the suspension was inoculated into a50 mL medium containing 20 g L^-1 ^glucose, 20 g L^-1^peptone and 10g L^-1 ^yeast extract. After 24 h. cultivation on a rotary shaker (180 rmin^-1^) at 28 °C, 50 mg of 16α, 17α-epoxyprogesterone (1) was added to the flask and the biotransformation was conducted for 24 h. under the same condition. The culture medium was analyzed by TLC.

*Separation and purification of the products*

The products were extracted from the medium by equal volume of ethyl acetatefor three times. After the evaporation of ethyl acetate in vacuum, white powder was obtained. After purification through silica gel column chromatography, two white products were obtained. Re-crystallization of products was performed and crystals were obtained, respectively 

*7β-hydroxy-16α, 17α-epoxyprogesterone (2)*

Whitecrystal.MS: Calcd. for C21H28O4**: **m/z 345.35 [M]^+^. 1H NMR (400 MHz, DMSO-*d*_6_) δ (ppm)): 5.64 (s, 1H); 4.70 (d, 1H,* J *= 6.4Hz); 3.90 (s, 1H); 3.15-3.13 (m, 1H); 2.45-2.38 (m, 3H); 2.35-2.12 (m, 2H); 2.01-1.89 (m, 5H); 1.63-1.54 (m, 4H); 1.53-1.42 (m, 1H); 1.41-1.25 (m, 1H); 1.22-1.00 (m, 7H); 0.89-0.88 (m, 1H). 13C NMR (DMSO): 205.5 (C(20)); 198.5 (C(3)); 168.7 (C(5)); 124.2 (C (4)); 73.5 (C(17)); 70.1 (C(7)); 61.1 (C(16)); 50.4 (C(6)); 44.4 (C(10)); 39.8 (C(2)); 39.6 (C(13)); 39.4 (C(8)); 38.1 (C(9)); 35.3 (C(1)); 34.1(C(12)); 31.4 (C(14)); 30.3(C(15)); 26.2 (C(11)); 20.4 (C(19)); 17.2 (C(21)); 15.3 (C(18)).

*7β,11α-dihydroxy-16α,17α-epoxyprogesterone (3)*

White crystal. MS: Calcd. for C21H28O5**: **m/z 361.39 [M]^+^.^ 1^H NMR (400 MHz, DMSO-*d*_6_) δ (ppm): 5.62 (s, 1H); 4.71 (d, 1H,* J* = 6.4 Hz); 4.40 (d, 1H, *J* = 6.8 Hz); 3.91 (s, 1H); 3.87-3.84 (m, 1H); 3.17-3.14 (m, 1H); 2.50-2.33 (m, 4H); 2.19-2.15 (m, 3H); 2.01-1.96 (m, 4H); 1.64-1.55 (m, 2H); 1.26-1.17 (m, 5H); 1.04-0.97 (m, 4H). 13C NMR (DMSO): 204.3 (C=O(20)); 198.0 (C(3)); 167.8 (C(5)); 123.5 (C(4)); 72.1 (C(17)); 68.8 (C(7)); 66.1 (C(11)); 60.3 (C(16)); 54.9 (C(9)); 42.5 (C(6))); 41.0 (C(2)); 39.1 (C(12)); 38.9 (C(10)); 38.7 (C(13)); 38.5 (C(8)); 35.8 (C(1)); 33.3 (C(14)); 29.2 (C(15)); 25.1 (C(19)); 17.5 (C(21)); 15.2 (C(18)).

## Results and Discussion

TLC was used to analyze the extracts from the culture of fungus. Figure 1 indicated the formation of two biotransformation products. Structures of the target products (2) and (3) were separated and confirmed by X-ray crystallography. The occurrence of hydroxylation at 7β and 7β, 11α of steroid nucleus was shown in Figure 2 and Figure 3.respectively.Characterization of (2) and (3) was further studied by MS and NMR spectroscopy. Characteristic chemical shifts of (1) (2) and (3) were summarized in Table1 and Table 2, respectively. Compared with substrate (1) the formation of the significant H signals of (2) at ppm values of 4.70 (d, 1H, 7-OH, *J *=6.4Hz) and 3.15-3.13(m, 1H, 7-CH) implied the 7β-hydroxylation of (1). In case of compound (3) the occurrence of new H signals at ppm values of 4.71 (d, 1H, 7-OH, *J *=6.4Hz). 3.17-3.14 (m, 1H, 7-CH). 4.40 (d, 1H, 11-OH, *J* = 6.8 Hz) and 3.87-3.84 (m, 1H, 11-CH) suggested the 7β, 11α-dihydroxylation of (1). Moreover, other chemical shifts of protons and carbons in methylene groups, methine groups, methyl groups, C=CH units and aryl rings were in reasonable ranges, and the data reflected the crystal structure of (2) and (3). Further MS data was in good accordance with (2) and (3). 

The biotransformation process of 16α, 17α-epoxyprogesterone (1) using P. decumbens in 24 h of incubation was also investigated. As shown in Table 3, products (2) was first detected in 2 hours. The yield of (2) increased and reached a plateau at around 44.5% after 12 h of incubation and then decreased to 22.8% after 18 h and continued going down. Product (3) was found after 6h incubation and its yield increased and finally reached around 79.7% after 24 h incubation. In control experiment, it was found that fermentation of compound (2) with P. decumbens also produced compound (3). This suggested during the biotransformation of (1), the first step is the conversion from (1) to (2) through 7β hydroxylation and the second is the conversion from (2) to (3) through 11α hydroxylation (Scheme 1). Penicillium species have been reported to carry out different types of steroid transformations, mainly including hydroxylation, lactonization and hydrogenation [14-16]. In this study, P. decumbens was found to selectively hydroxylation of steroid substrate with a different type of catalysis. Although microbial hydroxylation of steroids has been studied over the decades, P. decumbens can be a novel microorganism to perform the selective C7β and C11α hydroxylation of steroid. 

## Conclusions

Two new steroidal compounds were prepared from16α, 17α-epoxyprogesterone by *P. decumbens *for the first time. Their structures were characterized by 1H NMR, 13C NMR, X-ray crystallography and mass spectra. The biological activity of two new steroids will be studied in the near future.

## References

[1] Wu DX, Guan YX, Wang HQ, Yao SJ (2011). 11α-Hydroxylationof 16α,17-epoxyprogesterone by Rhizopusnigricans in abiphasic ionic liquid aqueous system. Bioresour. Technol..

[2] Shen LQ, Tang Y, Huang SY (2013). Synthesis of 25R-3β-chlorine-furosta-5, 20 (22)-dien-26-ol. Res. Chem. Intermed..

[3] Bhatti HN, Khera RA (2012). Biological transformations of steroidal compounds: A review. Steroids..

[4] Fragkaki AG, Angelis YS, Koupparis M, Tsantili-Kakoulidou A, Kokotos G, Georgakopoulos C (2009). Structural characteristics of anabolic androgenicsteroids contributing tobinding to the androgen receptor and to their anabolic and androgenic activities. Applied modifications in the steroidal structure. Steroids.

[5] Janeczko T, Dmochowska-Gładysz J, Kostrzewa-Susłow E, Białonska A, Ciunik Z (6651.). Biotransformations of steroid compounds by Chaetomium sp. KCH.

[6] Mernyák E, Kovács I, Minorics R, Sere P, Czégány D, Sinka I, Wölﬂing J, Schneider G, Újfaludi Z, Boros I, Ocsovszki I, Varga M ZupkóI (2015). Steroidal saponins from the leaves of Cordylinefruticosa (L) A Chev and theircytotoxic and antimicrobial activity. J. Steroid. Biochem .Mol. Biol..

[7] Wu Y, Li H, Zhang XM, Gong JS, Rao ZM, Shi JS, Zhang XJ, Xu ZH (2015). Efficient hydroxylation of functionalized steroids by Colletotrichumlini ST-1. J. Mol. Catal. B-Enzym..

[8] Nassiri-Koopaei N, Ali Faramarzi M (2015). Recent developments in the fungal transformationof steroids. Biocatal. Biotransform..

[9] Fernandes P, Cruz A, Angelova B, Pinheiro HM, Cabral JMS (2003). Microbial conversion of steroid compounds: recent developments. Enzyme. Microb. Technol..

[10] Gao JM, Shen JW, Wang JY, Yang Z, Zhang AL (2011). Microbial transformation of 3β-acetoxypregna-5,16-diene-20-one by Penicilliumcitrinum. Steroids.

[11] Feng M, Liao Z, Han L, Li J, Ye L (2014). Enhancement of microbial hydroxylation of 13-ethyl-gon-4-ene-3,17-dione by Metarhiziumanisopliae using nanoliposome technique. J. Ind. Microbiol. Biotechnol..

[12] Ma B, Shen Y, Fan Z, Zheng Y, Sun H, Luo J, Wang M (2011). Characterization of the inclusion complex of 16,17α-epoxyprogesterone with randomly methylatedβ-cyclodextrinin aqueous solution and in the solid state. J. Incl. Phenom. Macrocycl.Chem..

[13] Chen K, Tong WY, Wei DZ, Iang W (2007). The 11β-hydroxylation of 16,17α-epoxyprogesterone and the purificationof the 11β-hydroxylase from Absidiacoerulea IBL02. Enzyme. Microb. Technol..

[14] Zhang S, Liu PH, Zhao L, Liu XL (2014). Hydroxylation of Dehydroepiandrosterone by Penicilliumdecumbens ph-13. Lect. Notes. Electr. Eng..

[15] Holland HL, Dore S, Xu W, Brown FM (1994). Biotransformation of corticosteroids by Penicilliumdecumbens ATCC 10436. Steroids.

[16] Huang LH, Li J, Xu G, Zhang XH, Wang YG, Yin YL, Liu HM (2010). Biotransformation of dehydroepiandrosterone (DHEA) with Penicilliumgriseopurpureum Smith and Penicilliumglabrum (Wehmer) Westling. Steroids..

